# Developmental trajectory of long-lived plasma cells

**DOI:** 10.3389/fimmu.2025.1684210

**Published:** 2025-09-29

**Authors:** Takuya Koike, Wataru Ise

**Affiliations:** ^1^ Regulation of Host Defense Team, Division of Microbiology and Immunology, Center for Infectious Disease Education and Research, The University of Osaka, Osaka, Japan; ^2^ Department of Molecular Systems Immunology, University of Tokyo Pandemic Preparedness, Infection, and Advanced Research Center (UTOPIA), Tokyo, Japan; ^3^ Center for Advanced Modalities and Drug Delivery System (DDS), The University of Osaka, Osaka, Japan

**Keywords:** LLPC, bone marrow, antibody, niche, vaccine

## Abstract

Long-lived plasma cells (LLPCs), which continuously secrete antibodies, play a central role in humoral immunity and form the foundation of effective vaccine strategies. The anatomical segregation between the tissues where plasma cells are generated and where they are maintained suggests that both cell-intrinsic and extrinsic factors contribute to their longevity; however, the underlying cellular and molecular mechanisms remain largely unclear. In this review, we summarize recent advances in elucidating the regulation of plasma cell survival at both induction and effector sites. We particularly highlight potential LLPC precursors among newly generated plasma cells in secondary lymphoid tissues, and their subsequent maturation and differentiation into bona fide LLPCs within the bone marrow.

## 1 Introduction

Plasma cells are terminally differentiated effector cells responsible for the production of circulating antibodies, which confer protection against invading pathogens (1). Because of the half-life of antibodies is typically only a few days to weeks, long-term humoral immunity depends on the sustained presence of antigen-specific plasma cells.

While most plasma cells generated during infection or vaccination are generally short-lived, a subset acquires longevity and can persist for months, years, or even decades in human (2, 3). These cells, referred to as long-lived plasma cells (LLPCs), continuously secrete protective antibodies into the circulation (4–6). Durable antibody responses to certain viral infection or vaccines are thought to reflect the successful establishment of LLPCs within the bone marrow (5). Understanding the ontogeny and survival mechanisms of LLPCs may therefore help explain why the duration of antibody production varies widely across different vaccine (7) and, in turn, inform rational vaccine design.

**Figure 1:**
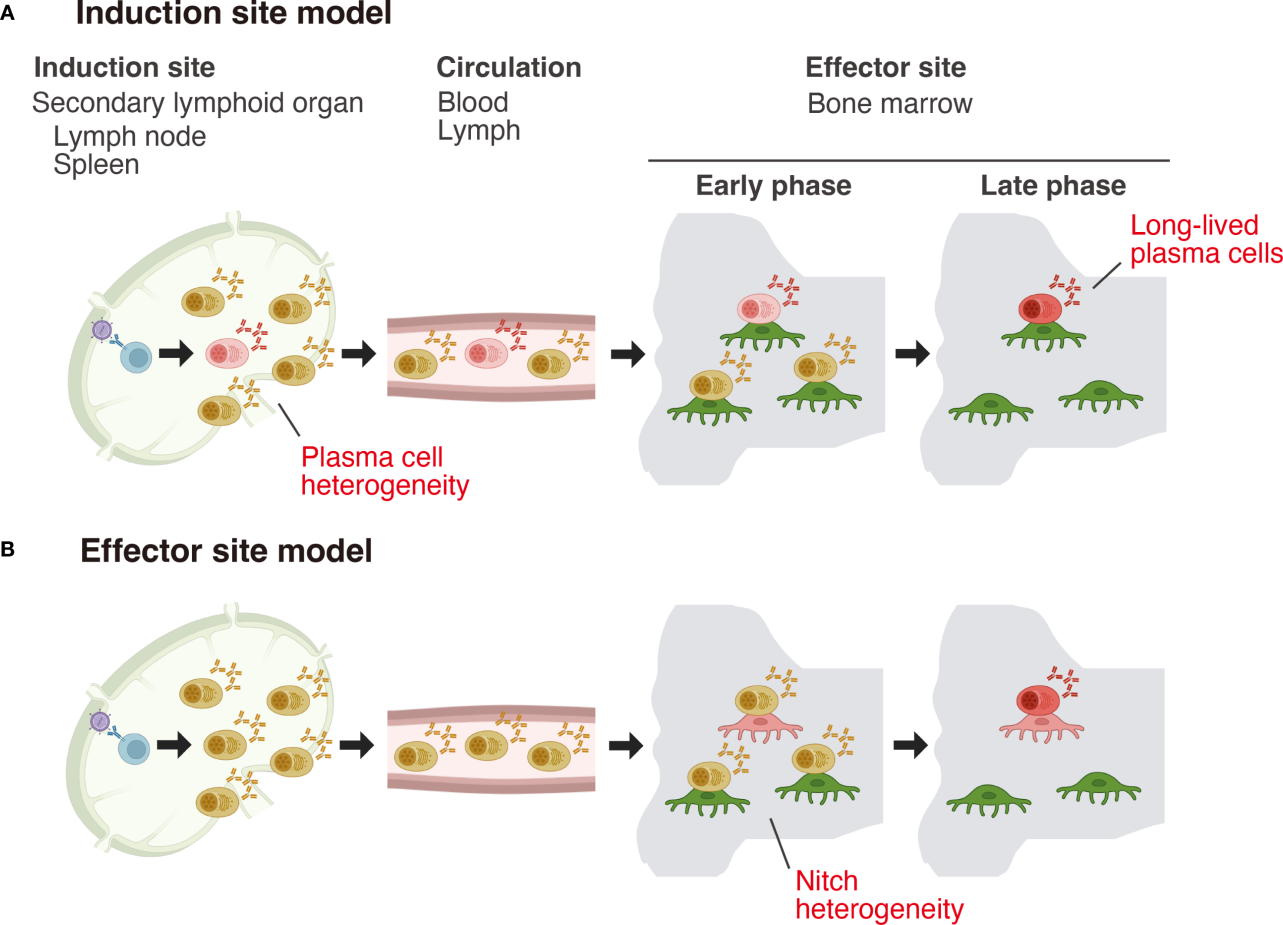
Two models for the acquisition of long-term survival capacity by plasma cells. **(A)** Induction site model: In this model, differences in plasma cell lifespan are programmed at the induction site, with the fate of becoming long-lived already imprinted at this stage. **(B)** Effector site model: In this model, plasma cells generated at the induction site are initially functionally uniform and stochastically migrate to the bone marrow. Within the bone marrow, a heterogeneous population of niche cells exists, and only plasma cells that interact with niche cells possessing high “nursing” capacity mature and acquire long-term survival potential.

It is well established that there is anatomical segregation between the sites where plasma cells are generated (induction sites) and the tissues where they home, secret antibodies, and persist long term (effector sites). Induction occurs almost exclusively in secondary lymphoid organs (SLOs), whereas LLPCs are maintained in a variety of anatomical locations (8). Two conceptual models have been proposed to explain how plasma cells acquire longevity (9) (**Figure 1**). The first, known as the effector site model, posits that newly formed short-lived plasma cells (SLPCs) migrate in response to the chemoattractants, such as CXCL12 (10), and those that fortuitously settle in survival-supportive niches, rich in pro-survival factors, mature into LLPCs and acquire extended longevity. In contrast, the second model proposes that heterogeneity in lifespan is programmed intrinsically at the induction site, with some plasma cells being fated to become long-lived immediately upon generation.

These extrinsic (niche-dependent) and intrinsic (cell-autonomous) determinants of plasma cell longevity are not mutually exclusive and likely act in concert. However, until recently, these models had not been formally tested due to the lack of suitable experimental tools. Advances in genetic models and single-cell sequencing technologies over the past several years are beginning to unravel the developmental trajectories of LLPCs, both at their induction sites and within their effector sites (11–13). In this review, we summarize recent progress in understanding the regulation of plasma cell longevity, with a particular focus on the characteristics of potential LLPC precursors found in the spleen and lymph nodes (as induction sites) and their subsequent turnover and survival within the bone marrow (as a representative effector site).

## 2 Plasma cell fate determination at the induction site

The prevailing view has long held that LLPCs or their precursors arise predominantly from germinal centers (GCs) in response to T cell-dependent antigens (14–16). This concept is supported by evidence that most bone marrow plasma cells generated in response to such antigens produce high-affinity, class-switched antibodies (17–19).

However, several findings have challenged this dogma. LLPCs are detectable in the bone marrow after T-independent immunization (20–22). A recent timestamping study, discussed later, revealed that while IgG and IgA LLPCs predominantly consist of somatically hypermutated clones generated after immunization or infection, IgM LLPCs are highly enriched in public clones that arise through T cell-independent differentiation, and display affinity for self-antigens or microbial-derived antigens (11). Direct evidence was further provided by Koike et al. who demonstrated that GC-independent plasma cells can persist in the bone marrow with similar decay kinetics to GC-derived plasma cells of the same antigen specificity (23). Together, these findings indicate that developmental trajectories leading to LLPCs are more diverse than previously assumed, and that GC experience is not a strict prerequisite for LLPC generation.

Two recent studies have identified subsets of plasma cells in secondary lymphoid organs (SLOs) that exhibit bone marrow tropism and likely serve as precursors to LLPCs. Manakkat Vijay et al. employed an NP-KLH immunization model and found that TIGIT^+^ plasma cells in the spleen give rise to bone marrow plasma cells (12). Adoptive transfer experiments using splenic CD138^+^ plasma cells isolated at various time points after immunization revealed that cells generated at a later stage (day 35) gave rise to more sustained serum NP-specific antibody responses and more effectively seeded the bone marrow compared to those generated earlier (day 21 or day 28). Combined single-cell RNA sequencing and B cell receptor (BCR) sequencing identified TIGIT^+^ splenic plasma cells as precursors of bone marrow plasma cells. The frequency of TIGIT-expressing plasma cells increased during the later stages of the immune response. Notably, only TIGIT^+^ (and not TIGIT^-^) plasma cells produced NP-specific antibodies in the serum and gave rise to NP-specific LLPCs in the bone marrow of recipient mice following adoptive transfer. Furthermore, TIGIT deficiency impaired the generation of both splenic and bone marrow plasma cells upon immunization, underscoring the functional importance of TIGIT in plasma cell biology. Mechanistically, TIGIT was shown to regulate plasma cell proliferation: TIGIT^+^ plasma cells exhibited enhanced proliferative capacity than their TIGIT^-^ counterparts. Consistently, TIGIT-deficient plasma cells showed reduced cell cycling, suggesting that TIGIT-dependent clonal expansion is critical for the migration of splenic plasma cells to the bone marrow survival niches.

In parallel, our group identified a subset of plasma cells in SLOs marked by high expression of integrin β7, which includes precursors of bone marrow plasma cells (13). Newly generated, antigen-specific IgG^+^ plasma cells in SLOs comprise both integrin β7^lo^ and β7^hi^ populations, whereas plasma cells that have recently arrived in the bone marrow are predominantly β7^hi^. Transcriptomic profiling and clonal tracing analyses revealed that β7^hi^ cells preferentially egress from SLOs to the bone marrow. Notably, bone marrow-tropic plasma cells were found in both GC-derived and GC-independent populations. This egress-prone subset expressed elevated levels of the transcription factor KLF2, a regulator of immune cell differentiation and migration (24). Conditional Klf2 deletion in plasma cells impaired their exit from SLOs and subsequent bone marrow migration. Although integrin β7 itself was unexpectedly dispensable, S1PR1 was identified as a key downstream effector of KLF2 in regulating plasma cell egress. This finding is consistent with previous studies showing that mice lacking S1pr1 in B cells, or treated with FTY720 (a functional S1PR1 antagonist), exhibit reduced numbers of IgG plasma cells in both the blood and bone marrow (25). Integrin β7^hi^ plasma cells also expressed elevated CD11b, which further contributed to their egress from SLOs.

Although the TIGIT^+^ (12) and integrin β7^hi^ subsets show slightly different induction kinetics, they likely overlap. Integrin β7^hi^ cells were Ki67^hi^, indicating that they are actively cycling, similar to TIGIT^+^ plasma cells. TIGIT is highly expressed in integrin β7^hi^ cells, and is downregulated in KLF2-deficient plasma cells (unpublished data), suggesting that TIGIT may be a downstream target of KLF2 in plasma cells. Functionally, Klf2 deficiency impaired the migration of plasma cells to the bone marrow following influenza vaccination, resulting in reduced durability of anti-HA IgG antibody responses and compromised protection against influenza infection (13). This aligns with findings in IgA^+^ plasma cells, where KLF2 controls migration from mesenteric lymph nodes (mLNs) to effector sites (26). In B cell-specific Klf2-deficient mice, IgA plasma cells accumulated in mLNs but were reduced in bone marrow, blood, spleen, and intestinal lamina propria. Transcriptomic analysis revealed KLF2-dependent regulation of migration-related genes, including integrins (Itgb7, Itgb1, ItgbM), selectins (L-selectin), chemokine receptors (Ccr9), and S1P receptors (S1pr1, S1pr3, S1pr4) in IgA^+^ plasma cells in the mLNs. The upstream signals that induce or sustain KLF2 expression in nascent plasma cells remain unclear. In mature B cells, the transcription factor Foxo1 has been implicated in the regulation of Klf2 gene expression: Foxo1 binds to the Klf2 promoter (27), and Klf2 mRNA levels are reduced in Foxo1-deficient B cells (28). Defining the molecular control of KLF2 in plasma cells will be critical for vaccine optimization.

**Figure 2:**
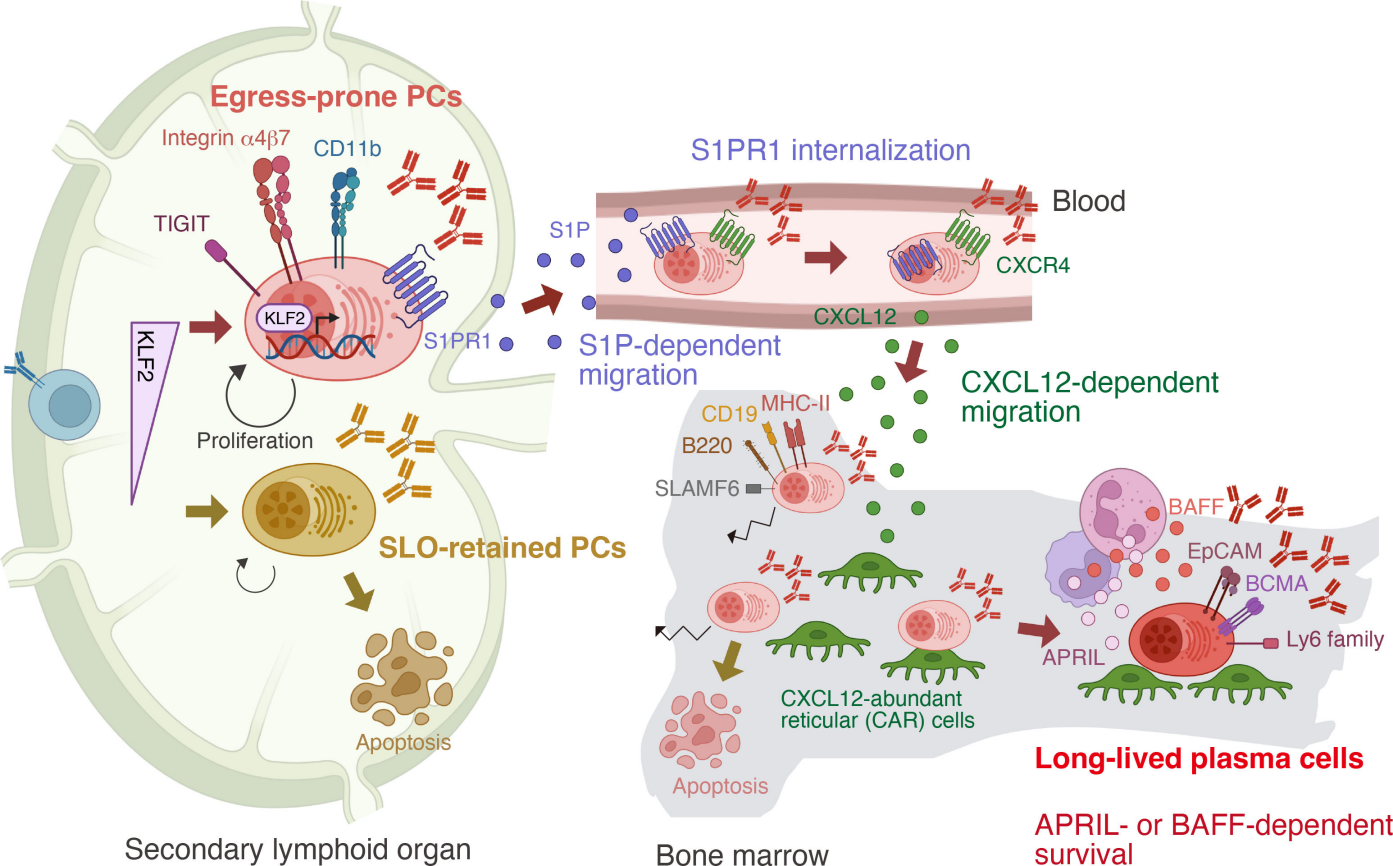
Stepwise differentiation toward long-lived plasma cells (LLPCs). In secondary lymphoid organs—the sites of plasma cell (PC) generation—PCs can be divided into two subsets based on expression of the transcription factor KLF2. Integrin β7^lo^ TIGIT^–^ PCs remain in the secondary lymphoid organs (SLOs), whereas Integrin β7^hi^ TIGIT^+^ PCs acquire the capacity to migrate to the bone marrow. This migration is driven by KLF2-mediated upregulation of S1PR1, which senses the gradient of sphingosine-1-phosphate (S1P). Upon entering the bloodstream, where S1P levels are high, ligand engagement leads to internalization of S1PR1. These PCs then respond to the chemokine CXCL12, which is produced in the bone marrow, and migrate into the bone marrow. Only a fraction establish long-term residency and mature into LLPCs, which can be distinguished from recently arrived PCs by distinct surface marker expression. Within bone marrow niches, LLPCs are sessile and receive survival support from factors such as APRIL and BAFF produced by niche cells.

Our working model, illustrated in **Figure 2**, proposes that plasma cell fate is determined early by KLF2 expression. Upon vaccination or infection, a KLF2-dependent, egress-prone subset characterized by high integrin β7, CD11b, S1PR1, and TIGIT, is induced in SLOs. Cells lacking sufficient KLF2 fail to exit and die *in situ*. S1PR1 is essential for plasma cell egress into the bloodstream via the S1P gradient, but subsequent S1PR1 internalization is required for entry into the bone marrow (29). Once in circulation, plasma cells home to the bone marrow in response to the chemoattractant CXCL12 (10). Upon lodging in available survival niches, they gradually mature into LLPCs, as discussed below, by receiving critical survival cues, such as APRIL or BAFF, from the local microenvironment (30).

## 3 Timestamping analysis of plasma cell

Addressing the question of LLPC longevity has been a challenging task. LLPCs are not only extremely rare, but they also lack definitive phenotypic markers that distinguish them from SLPCs. Previous studies have primarily relied on the detection of antigen-specific plasma cells or the measurement of antigen-specific antibodies in the serum to evaluate the persistence of plasma cells following immunization (5, 31). However, because plasma cells are continuously generated as long as the immune response persists, the resulting plasma cell pool comprises a mixture of newly generated and pre-existing cells (32). Another strategy has involved labeling newly formed plasma cells with thymidine analogs such as BrdU and tracking them overtime (4, 20). While this method allows for temporal discrimination of plasma cell populations, it is incompatible with downstream molecular analyses, such as transcriptomic profiling, due to technical limitations associated with BrdU detection.

A major methodological breakthrough came with the development of a genetic plasma cell fate-mapping system, commonly referred to as plasma cell “timestamping”. Five research groups independently generated transgenic mouse lines expressing tamoxifen-inducible Cre recombinase (CreERT2) under the control of plasma cell-specific genes, such as *Ighj* or *Prdm1* (11, 23, 33–35). When combined with a Cre-dependent reporter allele (e.g., Rosa26-LSL-tdTomato), administration of tamoxifen induces permanent expression of a reporter specifically in plasma cells present at the time of treatment. As a result, these timestamped cells can be tracked over time, while plasma cells generated after tamoxifen administration remain reporter-negative. This approach enables researchers to follow the fate of plasma cells present at a defined time point. Reporter-positive cells that persist for several months after tamoxifen treatment can be considered bona fide LLPCs, while the reporter-negative population contains a heterogeneous mixture of newly generated plasma cells of varying ages.

A series of timestamping studies have indeed yielded several novel insights into plasma cell biology, the details of which have been comprehensively reviewed elsewhere (36). The key points can be summarized as follows. First, homeostatic plasma cells are continuously replenished by newly generated populations, some of which progressively differentiated into LLPCs (23, 37). Second, following immunization with a model antigen, LLPCs accumulate in the bone marrow at a relatively constant rate from the early stages of the immune response, with no apparent bias toward the later phases (23, 33). Third, bone marrow plasma cells are highly heterogeneous in terms of maturation status and immunoglobulin isotype, with LLPCs comprising transcriptionally distinct subsets expressing IgA, IgM, or IgG (11, 34, 37). Fourth, LLPCs are enriched for a unique gene expression signature and display a distinct pattern of surface markers, enabling their distinction from bulk plasma cells or SLPCs (11, 34, 37). Fifth, plasma cells gradually lose motility within the bone marrow microenvironment, with LLPCs exhibiting a sessile, immobilized state within their survival niches (23, 34).

## 4 Regulation of plasma cell survival in the bone marrow

Timestamping approaches have provided new insight into one of the long-standing questions in the field: how newly generated plasma cells persist in the bone marrow despite the presence of pre-existing, established plasma cells. Two mutually exclusive models have been proposed to explain this phenomenon. The saturable niche model posits that the bone marrow contains a finite number of survival niches; newly formed plasma cells must compete for these limited sites, displacing resident cells to achieve long-term persistence (38). In contrast, the unlimited niche model suggests that new plasma cells occupy unfilled or newly created niches without directly competing with existing cells.

Recent timestamping studies appear to favor the latter model. Koike et al. reported no significant difference in bone marrow persistence between early (pre-GC) and late (post-GC) plasma cells, indicating that the timing of arrival does not dictate survival (23). Moreover, the absolute number of homeostatic plasma cells in the bone marrow gradually increases with age, indicating that niche capacity is not saturated over time (23). Similarly, Robinson et al., found that altering the influx of newly generated plasma cells, either increasing or decreasing their numbers, did not affect the turnover rate of pre-existing, timestamped plasma cells (37). Collectively, these findings suggest that plasma cell turnover in the bone marrow is governed primarily by intrinsic factors rather than by direct competition for limited niche resources, at least under homeostatic conditions or following immunization with model antigens and adjuvants. Whether different types of antigens and adjuvants can alter the turnover of bone marrow plasma cells remains an open question.

The possibility that bone marrow survival niches are heterogeneous in their capacity to support plasma cell longevity (**Figure 1**) also remains to be fully addressed. Jing et al. found that LLPCs are more frequently found in clusters than bulk plasma cells (34). This clustering depends on hematopoietic-derived APRIL (39), as acute APRIL inhibition disrupts these clusters and mobilizes plasma cells (30). Megakaryocytes are a known source of APRIL in the bone marrow (40), and their activation by thrombopoietin (TPO) administration has been shown to enhance the longevity of vaccine-induced antibody titers (41), indicating that megakaryocytes form a key component of LLPC survival niches. Furthermore, recent transinteractome analyses suggest that distinct subsets of plasma cells may depend on different cellular interaction partners, implying that their retention within the bone marrow is mediated by more diverse molecular mechanisms than previously appreciated (42). These observations point to the existence of functionally specialized microenvironments within the bone marrow that differentially regulate plasma cell survival. Fully elucidating the nature and organization of LLPC-supportive niches will require more refined approaches, such as spatial transcriptomics.

## 5 Conclusion and outstanding questions

Accumulating evidence has begun to reveal the developmental trajectories to LLPCs. A key emerging concept is that plasma cell longevity is “imprinted” early during their generation at the induction site. Depending on BCR signal and T cell help, activated B cells give rise to a small fraction of pre-plasma cells committed to the plasma cell lineage (43, 44). Their progeny, however, remain heterogenous, and only a fraction possesses the potential to become BM-resident LLPCs. These LLPC precursors are further shaped by turnover within extrinsic survival niches, gradually acquiring the features required for longevity. Collectively, both early imprinting at the induction site and subsequent refinement within effector sites cooperate to establish bona fide LLPCs.

Several key questions remain to be answered. First, the mechanisms governing the generation of LLPC precursors, such as TIGIT^+^ cells (12) or Integrin β7^+^ cells (13) at the induction site require further clarification. A central issue is which signals induce these precursors, and whether they are already specified at the pre-plasma cell stage or instead emerge only after plasma cell identity has been established. Moreover, it remains unresolved whether analogous precursors exist across other antibody isotypes or arise within distinct anatomical induction sites. Second, it remains unclear whether different forms of antigen and/or adjuvants influence the generation of LLPC precursors or their cell-intrinsic longevity. This may explain why durability of antibody response varies among vaccines (7) and addressing it could guide the design of next-generation vaccines capable of providing lifelong protection. Third, the precise characteristics and potential heterogeneity of bone marrow survival niches need to be defined. High-resolution single cell spatial omics will be indispensable for this task. Lastly, the signals and molecular pathways that drive LLPC maturation within these niches remain to be identified. The contribution of candidate regulators, including APRIL, BAFF (45), metabolites (46, 47), or adhesion molecules (39, 48), to LLPC maturation and survival needs to be determined. Clarifying these outstanding questions will be critical not only for the rational design of vaccines capable of inducing long-lasting humoral immunity, but also for the development of therapeutic strategies targeting autoimmune diseases in which chronically produced antibodies drive pathology.
